# Ohmic Contact to n-GaN Using RT-Sputtered GaN:O

**DOI:** 10.3390/ma16165574

**Published:** 2023-08-11

**Authors:** Monika Maslyk, Pawel Prystawko, Eliana Kaminska, Ewa Grzanka, Marcin Krysko

**Affiliations:** Institute of High Pressure Physics, Polish Academy of Sciences, Sokolowska 29/37, 01-142 Warsaw, Poland; pprysta@unipress.waw.pl (P.P.); ekaminska@unipress.waw.pl (E.K.); ewa.grzanka@unipress.waw.pl (E.G.); krysko@unipress.waw.pl (M.K.)

**Keywords:** GaN, oxygen, magnetron sputtering, ohmic contact, regrown contact, sub-contact layer, doping, ammonothermal GaN

## Abstract

One of the key issues in GaN-based devices is the resistivity and technology of ohmic contacts to n-type GaN. This work presents, for the first time, effective intentional oxygen doping of sputtered GaN films to obtain highly conductive n^+^-GaN:O films. We have developed a novel and simple method to obtain these films. The method is based on the room temperature magnetron sputtering of a single crystal bulk GaN target doped with oxygen. The n^+^-GaN:O films exhibit a polycrystalline structure with a crack-free surface and a free electron concentration of 7.4 × 10^18^ cm^3^. Ohmic contact to GaN:Si with n^+^-GaN:O sub-contact layer achieves specific contact resistance of the order of 10^−5^ Ωcm^2^ after thermal treatment. The obtained results are very promising for the development of the technology of a whole new class of ohmic contacts to n-GaN.

## 1. Introduction

One of the key issues in research and development in areas such as optoelectronics and high-frequency and high-power electronics remains the formation of reliable ohmic contacts to GaN-based structures and devices [1,2,3]. This issue is of interest because of unresolved problems with the degradation of these ohmic contacts, difficulties in doping GaN with Si and the limitations of the technologies used for their fabrication.

The most common metallization scheme for n-type GaN consists of (from bottom to top) low work function metals Ti (4.33 eV) and Al (4.28 eV), an antidiffusion barrier (Ni, Mo, TiN, TaSiN) and capping layer (Au) [4]. The ohmic behavior of this stack is formed by high temperature treatment in the range of 650–850 °C, mainly 850 °C [4]. The dominant mechanism of formation of this ohmic contact is based on the out-diffusion of nitrogen from the subsurface region in GaN to the metal/GaN interface, creating nitrogen vacancies V_N_ that act as donors [5]. However, diffusion, mixing and reactions between the semiconductor and metals occur simultaneously, resulting in severe increases in roughness, contact resistance and inhomogeneity [6]. These changes also cause a lot of damage to the devices.

As an alternative to the method described above, the selective ion implantation of donor dopants [7,8] or epitaxial growth (regrowth) of a highly-doped n^+^-GaN sub-contact layer in the recessed contact regions [9,10,11] are used to form ohmic contacts. To locally increase the donor doping in these regions, Si atoms are the element of choice, with a high solubility in GaN of the order of 10^20^ cm^−3^ and a carrier concentration in the range 10^17^–10^19^ cm^−3^ [12]. The donor state introduced by Si dopants is about 20 meV below the minimum of the conduction band, so in Si-doped GaN, the free carrier concentration is similar to the dopant concentration [13]. However, for Si dopant concentrations higher than 10^19^–10^20^ cm^−3^, the degradation of material quality and electron concentration occurs because tensile strain also limits the maximum thickness of Si-doped GaN films [12,14]. To overcome this problem, other dopants such as Se [15] and Ge [16] have been investigated.

Despite the fact that the regrowth process is currently the method that allows the lowest ohmic contact resistance to be achieved with n-GaN, this process is associated with a number of technological difficulties and limitations. The regrowth of n^+^-GaN:Si sub-contact layers through metal organic chemical vapor deposition (MOCVD) and molecular beam epitaxy (MBE) are carried out at high substrate temperatures, typically above 600 °C [17]. A regrowth process could be problematic, as the wafers require additional handling and transport to the MOCVD or MBE facility, leaving the conventional processing lab. It may also not be cost effective to use specialized and expensive MOCVD/MBE equipment.

Magnetron sputtering, as a potential method for the n^+^-GaN sub-contact layer growth, has gained increasing attention in recent years due to its simplicity, scalability and multifunctionality. It allows the control of many process parameters over a wide range, thereby influencing the film properties. Crystalline structure, stoichiometry and impurity concentration are strongly dependent on gas pressure, sputtering atmosphere composition and substrate temperature. Several sputtering techniques have been developed. GaN thin films are mainly deposited from Ga targets in RF or pulsed mode, in N_2_/Ar ambient gas, usually without buffer layers on a substrate and with Si as dopant, at temperatures typically around 500 °C [14,17,18,19]. GaN thin films grown using magnetron sputtering have a predominantly polycrystalline structure with the dominant GaN 0002 plane. Shinoda et al. [19] have obtained a decrease in full width at half maximum (FWHM) for the GaN 0002 plane with decreasing N_2_ composition ratio from 40% to 6%. The as-grown electronic-grade GaN films sputtered from a Ga target in a N_2_/Ar atmosphere on Al_2_O_3_ c-plane substrates by Junaid et al. were characterized by dislocation density ≤ 10^10^ cm^−2^ [20]. In addition, MOCVD and MBE are not compatible with the conventional lift-off process used to form a patterned metallization on a substrate. Conventional photoresists can only be used at relatively low substrate temperatures, as they typically reflow at 100–150 °C. Magnetron sputtering allows deposition at low temperatures due to the energies of the sputtered atoms of a few eV.

To alleviate some of the difficulties described above, we present both a novel ohmic contact to n-GaN with a n^+^-GaN:O sub-contact layer deliberately doped with oxygen and a method of obtaining this layer. We used room temperature magnetron sputtering from a bulk GaN target containing oxygen in dopant concentration. Oxygen is a donor impurity in GaN thin films with a shallow energy level that contributes to the non-intentional electron conductivity of undoped GaN films [12,13]. As we can see from the literature review, oxygen has mostly been studied as a non-intentional impurity [21,22,23]. In our work, intentionally oxygen-doped n-GaN films and ohmic contacts were investigated structurally, optically, electrically and with circular TLM characterization.

## 2. Materials and Methods

**Figure 1:**
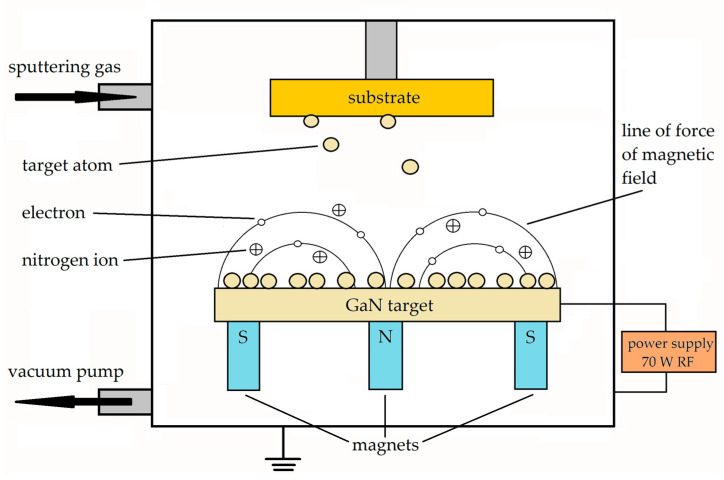
Scheme of the magnetron sputtering process for n-GaN:O thin film deposition.

GaN:O thin films were deposited via magnetron sputtering in a Korvus Technology Hex-80 system (Figure 1). The processes were performed using a 5 mm thick and 2″ diameter n-type bulk single crystal of GaN:O as the target, prepared via the basic ammonothermal method and containing an oxygen concentration of 1.2 × 10^19^ cm^−3^ [24]. The substrates used for deposition were c-plane sapphire Al_2_O_3_, semi-insulating (SI) GaN and n-type GaN 0002 epitaxial MOCVD templates on sapphire. The top of the n-GaN layers was 1 µm thick n-GaN:Si with a carrier concentration of 7 × 10^16^ cm^−3^, as determined through C-V measurements. This low carrier concentration value allows the influence of the sub-contact n^+^-GaN:O layer on the ohmic contact performance and properties to be more precisely defined. Prior to deposition, substrates were sequentially cleaned by soaking in Piranha solution at 120 °C for 3 min and in Piranha solution in an ultrasonic bath for 3 min, then rinsed with deionized water and dried via N_2_ blowing. Substrates were placed in the sputter chamber at a distance of 12 cm from the target and were not intentionally heated during the process. The initial vacuum level of ˂1 × 10^−2^ Pa is crucial in the doping process as it can influence the concentration of non-intentional elements in the film. Magnetron sputtering was performed in an atmosphere of 100% N_2_ atmosphere at a total pressure of 0.23 Pa and in RF mode (13.56 MHz) at 70 W power. GaN:O films of 337 nm thickness on the SI-GaN substrate and 50 nm thickness on other substrates (sapphire and GaN/sapphire) were deposited at a growth rate of about 0.34 Å/s.

After deposition, the surface of GaN:O films on GaN epitaxial layers was cleaned by soaking in diluted HF:HCl solution at room temperature for 60 s followed by rinsing with deionized water and drying by N_2_ blast. This step removes native oxide from the GaN:O surface and is necessary because such native oxide introduces an additional barrier potential at the metallization/GaN:O interface. The GaN:O films were then patterned in a circular transmission line method (c-TLM) using lift-off negative photolithography. The radius of the circular inner region *r* was 40 µm and 60 µm. The contact spacing *d* was in the range of 4.5–60 µm, and the films were rinsed in HF:HCl:DI solution, as described above. The metallization layers of Ti/Al/Ti/TiN 20/60/15/10 nm were deposited via magnetron sputtering in the same Korvus system. Lift-off was performed, followed by rinsing with deionized water and drying with an N_2_ blast. The ohmic contacts were then annealed at 850 °C for 60 s in a nitrogen flow using a Jipelec JetFirst rapid thermal processing system.

GaN:O thin film on sapphire Al_2_O_3_ substrate were characterized through X-ray diffractometry (XRD) in θ–2θ mode and through low angle grazing incidence X-ray reflectometry (XRR) using a Malvern Panalytical Empyrean diffractometer. XRD measurements were performed over a wide range of 10–110 degrees to identify crystalline materials. From XRR measurements, the density and thickness of each layer and the surface roughness of GaN:O were calculated by fitting the measured data with a theoretical model. Surface topography and roughness were investigated via atomic force microscopy (AFM) using 1 × 1 µm, 3 × 3 µm and 10 × 10 µm scans on a Veeco Dimension 3100 tool. Optical characterization was carried out using a Fourier transform infrared (FTIR) spectrometer operating in rapid scan mode with a KBr beam splitter, a DLTGS pyroelectric photodetector and a 3 mm diameter metal aperture. Reflection spectra were obtained at 300 K, 1.5 kPa pressure and in the 4000–400 cm^−2^ wave number range. The performance and properties of the ohmic contacts were investigated at 300 K through I-V characterization using a two-point probe and a Keithley 2410 Source-Measure Unit (SMU) and LabTracer 2.9 SourceMeter integration software. Measurements were made from −1 V to +1 V with a 0.013 step and the subsequent extraction of electrical parameters. As-deposited and annealed contacts were analyzed.

## 3. Results and Discussion 

**Figure 2:**
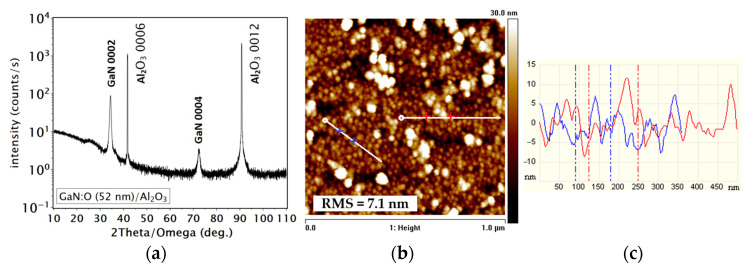
X-ray diffraction (XRD) pattern of GaN:O film on sapphire Al_2_O_3_ substrate (**a**) and atomic force microscopy (AFM) surface image of GaN:O film on sapphire Al_2_O_3_ substrate, 1 × 1 µm scan (the red and blue + indicate red and blue dashed lines in the (**c**)) (**b**), with profile lines of the surface (**c**).

X-ray diffraction on the sapphire c-plane substrate revealed the crystal structure of the GaN film (Figure 2a). The diffractogram shows a polycrystalline structure with diffraction peaks associated with GaN 0002 and GaN 0004 reflections with relatively high intensities as well as with substrate Al_2_O_3_ 0006 and Al_2_O_3_ 0012 peaks, according to the ICCD database. The GaN 0002 peak is slightly shifted to lower angles (~0.1 deg.), which may be due to an increase in the lattice parameters characteristics of nanocrystals rather than to the oxygen doping and lattice mismatch of the film and substrate. The XRD pattern did not show any additional peaks associated with other phases. The XRD suggests a crystallite size of about 15 nm. The crystal structure of the GaN:O film is influenced by the substrate structure, with the same c-plane orientation. The possible explanation for the obtained GaN:O crystal structure may be the features of magnetron sputtering itself, i.e., the energies of the sputtered atoms of a few eV [25], relatively high target-substrate distance during the process (12 cm) and sputtering from a high purity crystalline target [26]. The high kinetic energy of the sputtered atoms results in high atomic mobility on the substrate, which resulted in an increase in the mean free path and ultimately enhanced the nucleation and crystallization of the film. This behavior is further enhanced by the relatively low growth rate of the GaN:O film. The plasma species are mainly close to the target, so the growing film is less exposed to the point defect-producing species [27]. It has been shown in the literature that GaN layers deposited by magnetron sputtering can exhibit high structural quality even on sapphire substrates, but the process has mostly been performed at elevated temperatures and under Ga-rich conditions [19,20,28]. A more detailed insight into the growth mechanism of GaN:O films could be obtained via plasma studies, i.e., Langmuir probe or optical emission spectroscopy (OES) [29].

**Figure 3:**
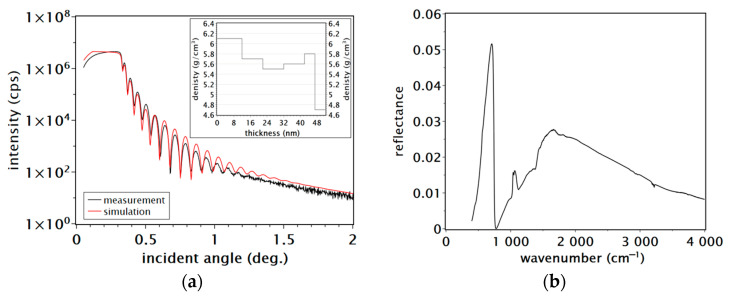
(**a**) X-ray reflectometry (XRR) pattern of GaN:O film on sapphire substrate. The inset graph shows the density distribution as a function of film thickness from the substrate/film interface (0 on the 0X axis) to the film surface (52 nm on the 0X axis); (**b**) typical reflection Fourier transform infrared (FTIR) spectrum of the GaN:O film on the SI-GaN substrate.

The surface morphology of the GaN:O film on the sapphire substrate shows a low root mean square (RMS) roughness of about 7 nm. The typical AFM surface image and profile lines are shown in Figure 2b,c, respectively. The macroscopic surface morphologies were bubble-like with anisotropic distribution, without cracks or pits. The bubble-like surface morphology may be related to the low subsurface density of the GaN:O film, as revealed by XRR measurements. The inset graph in Figure 3a shows the density distribution of the GaN:O film as a function of its thickness. The sub-surface region of 5 nm shows a very low density of about 4.7 g/cm^3^. The density of the GaN:O film near the substrate surface is close to the bulk GaN density of 6.14 g/cm^3^, i.e., 6.1 g/cm^3^, then decreases, increases and decreases again as a function of the film thickness. The simulation model shows good agreement with the experimental data (Figure 3a). A GaN:O film density lower than its bulk value may indicate the presence of voids or an amorphous phase.

FTIR measurements were used to determine the carrier concentration in the GaN:O film. The infrared reflectance spectrum of a n-type semiconductor is described by the Lorentzian oscillator model [30,31]. In this model, the dielectric function and the complex refractive index allow us to obtain the plasma frequency *v_p_* and then the free electron concentration *N*:

(1) $$N=v_{p}^{2}Km^{*}\in_{\infty },$$

where *v_p_* is the plasma frequency (plasma edge), *K* is the const = 1.115 × 10^15^ m^−1^, m* is the electron effective mass in GaN (0.22) and $\in_{\infty }$ is the high frequency dielectric constant (5.5). The plasma frequency for the reflectivity minimum in the FTIR spectrum for the GaN:O film is 741 cm^−1^ (Figure 3b), so the calculated carrier concentration *N*, according to Equation (1), is 7.41 × 10^18^ cm^−3^, which corresponds to half the initial oxygen content in the target material. However, it should be noted that the GaN LO phonon frequency peak in the low temperature luminescence spectrum is at 741 cm^−1^ (at 92.5 meV) [32]. Thus, the energy position of the minimum in the reflection spectra is similar to the longitudinal optical phonon of GaN. The calculated value of the free electron concentration in the GaN:O film confirms the effectiveness of intentional doping with oxygen and the presence of oxygen atoms substituting nitrogen atoms (O_N_) in the GaN lattice, forming shallow donor states [12].

**Figure 4:**
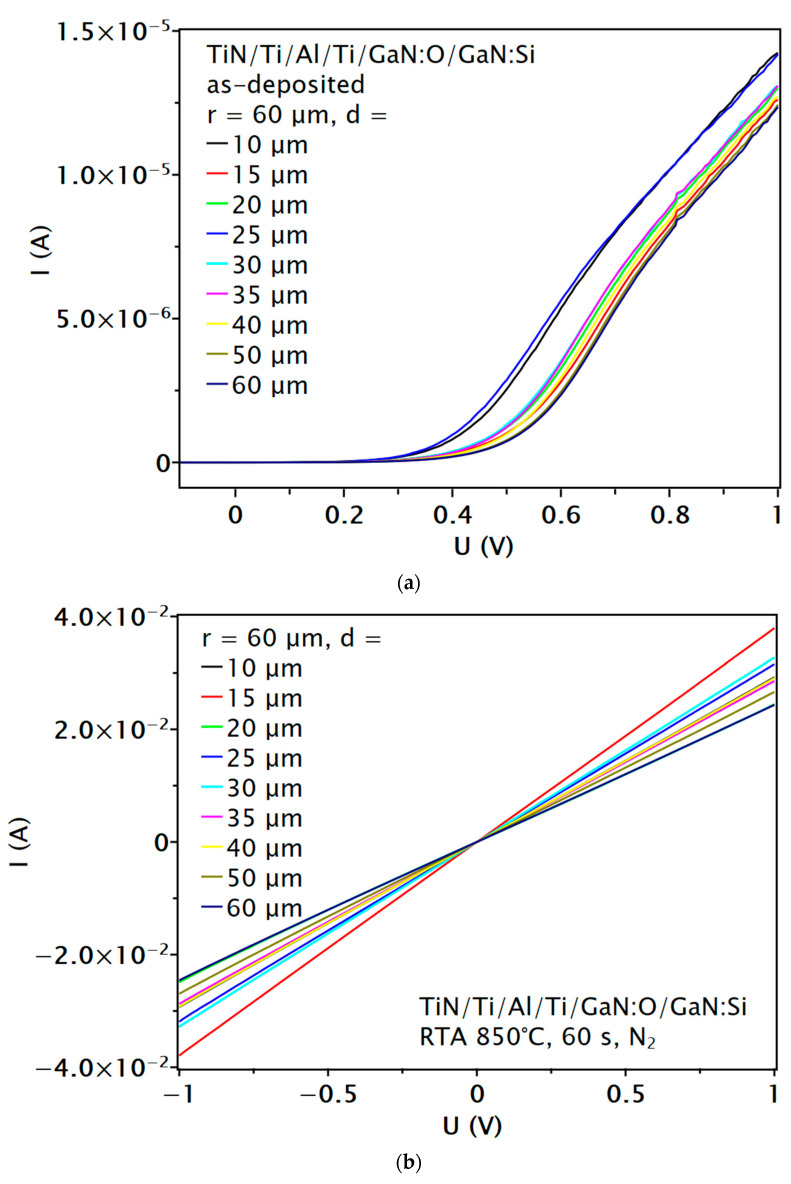
Typical two-point probe I-V plots of the circular transmission line method (c-TLM) patterns for the ohmic contact to n-GaN:Si with sub-contact n^+^-GaN:O layer as-deposited (**a**) and annealed (**b**).

**Figure 5:**
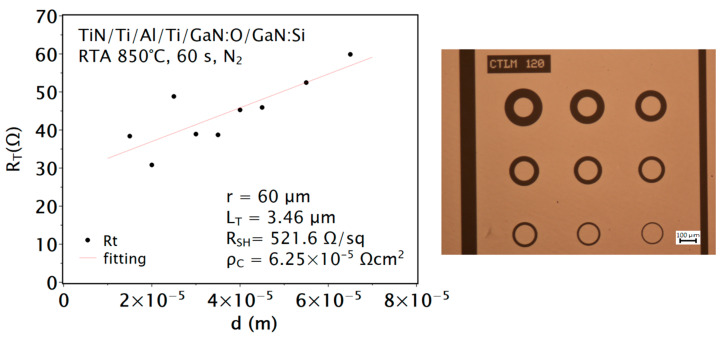
Resistance *R_T_* measured as a function of contact spacing *d* with extracted parameters. The drawing on the right shows the geometry of the contacts.

The as-deposited ohmic contacts to n-GaN:Si with sub-contact n^+^-GaN:O layer were non-linear (Figure 4a). There are not many reports in the literature describing ohmic contacts to n-GaN without post-deposition annealing or annealing at relatively low temperatures. In addition, such works are only concerned with highly doped semiconductors [33,34]. In this work, annealing at 850 °C in a nitrogen atmosphere resulted in an ohmic character of the contact (Figure 4b). The values of the calculated specific contact resistance *ρ_c_* = 6.25 × 10^−5^ Ωcm^2^, sheet resistance *R_SH_* = 521.6 Ω/sq and transfer length *L_T_* = 3.46 µm (Figure 5) [35] are similar to those reported in the literature for contacts to n-GaN with or without n^+^-GaN:Si sub-contact layer and were annealed under the same conditions, including those fabricated using methods other than magnetron sputtering. The formation mechanism of this ohmic contact is thought to be similar to that of conventional Ti/Al-based ohmic metallization on GaN:Si layers. Annealing, which produces N-vacancies and thus a higher donor concentration in the sub-contact region, improves the ohmic contact formation.

It is worth noting that the presence of oxygen in the n^+^-GaN:O sub-contact layer did not cause the formation of barrier oxides during metallization annealing. Such a change could result in a high resistance contact.

## 4. Conclusions

Oxygen-doped GaN films have been deposited by room temperature magnetron sputtering from an oxygen-containing monocrystalline bulk GaN target. The n^+^-GaN:O films exhibit a polycrystalline structure with a dominant GaN 0002 plane, a surface morphology without cracks or pits and an inhomogeneous density lower than that of bulk GaN. The intentional doping of GaN with oxygen results in high n-type conductivity with an electron concentration of around 7.4 × 10^18^ cm^−3^. The results obtained, particularly the ohmic contact to n-GaN:Si with the n^+^-GaN:O sub-contact layer, proved the effectiveness and validity of the intentional doping of n-GaN with oxygen. These also demonstrate the potential of this approach for developing low resistance and crack-free ohmic contacts to n-GaN.

## Data Availability

All data are contained within this article.
